# KIFC1-Like Motor Protein Associates with the Cephalopod Manchette and Participates in Sperm Nuclear Morphogenesis in *Octopus tankahkeei*

**DOI:** 10.1371/journal.pone.0015616

**Published:** 2010-12-20

**Authors:** Wei Wang, Jun-Quan Zhu, He-Ming Yu, Fu-Qing Tan, Wan-Xi Yang

**Affiliations:** 1 Faculty of Life Science and Bioengineering, Ningbo University, Ningbo, China; 2 The Sperm Laboratory, College of Life Sciences, Zhejiang University, Hangzhou, China; 3 The First Affiliated Hospital, College of Medicine, Zhejiang University, Hangzhou, China; University of Hong Kong, Hong Kong

## Abstract

**Background:**

Nuclear morphogenesis is one of the most fundamental cellular transformations taking place during spermatogenesis. In rodents, a microtubule-based perinuclear structure, the manchette, and a C-terminal kinesin motor KIFC1 are believed to play crucial roles in this process. Spermatogenesis in *Octopus tankahkeei* is a good model system to explore whether evolution has created a cephalopod prototype of mammalian manchette-based and KIFC1-dependent sperm nuclear shaping machinery.

**Methodology/Principal Findings:**

We detected the presence of a KIFC1-like protein in the testis, muscle, and liver of *O. tankahkeei* by Western Blot. Then we tracked its dynamic localization in spermatic cells at various stages using Immunofluorescence and Immunogold Electron Microscopy. The KIFC1-like protein was not expressed at early stages of spermatogenesis when no significant morphological changes occur, began to be present in early spermatid, localized around and in the nucleus of intermediate and late spermatids where the nucleus was dramatically elongated and compressed, and concentrated at one end of final spermatid. Furthermore, distribution of the motor protein during nuclear elongation and condensation overlapped with that of the cephalopod counterpart of manchette at a significant level.

**Conclusions/Significance:**

The results support the assumption that the protein is actively involved in sperm nuclear morphogenesis in *O. tankahkeei* possibly through bridging the manchette-like perinuclear microtubules to the nucleus and assisting in the nucleocytoplasmic trafficking of specific cargoes. This study represents the first description of the role of a motor protein in sperm nuclear shaping in cephalopod.

## Introduction

Spermatogenesis is a highly-ordered developmental process beginning in the testis with proliferation and differentiation of spermatogonia, incorporating mitotic and meiotic divisions, and ending up with spermiogenesis which witnesses dramatic structural, functional and morphological changes transforming spermatids towards mature spermatozoa [1]–[3]. Among all the cytological changes occurring during this process, biogenesis of the lysosome-like acrosome, elongation and condensation of the nucleus, and formation of the motile flagellum are of prime significance [4]. The normal morphogenesis of sperm nucleus, or nuclear shaping, is especially important for the viability of sperm because the appropriately streamlined nucleus is an indispensable structure of mature sperm accommodating paternal genetic materials vital for propagation of species.

In many vertebrates, the morphological transformations involved in the differentiation of spermatid are dependent on dedication of various cellular elements including cytoskeleton network and associated molecular motor proteins [5]–[8]. As an important type of cytoskeleton, microtubule is essential to several morphogenesis events including sperm nuclear shaping [9], [10]. At specific stages during spermiogenesis, a bundle of microtubules in the distolateral region of cytoplasm will transiently assemble around the nucleus to form a special structure called the manchette, which is believed to be indispensable for acquisition of the final nuclear morphology [7], [11], [12] and delivery of molecules to centriole and tail [13]–[15]. Kinesin is a superfamily of motor proteins that walk along microtubules to sort and transport various cellular cargoes to different destinations [16]–[18]. Many kinesin members have been identified from testis with suggested roles in multiple cellular aspects of spermatogenesis [19]–[22]. KIFC1 belongs to the kinesin-14 subfamily, a group of highly related C-terminal motor proteins with divergent tail domains [16], [19], [21], [23]. During rat spermiogenesis, KIFC1 is involved in the transport of proacrosomal vesicles from Golgi apparatus to the forming acrosome [24]. The protein also associates with a nuclear pore protein-containing complex on the nuclear envelope while moving along manchette microtubules and contributes to the generation and transmission of force needed for the shaping of nucleus [25].

The biological organization at the cellular and molecular level during spermatogenesis is exposed to an exceptionally fast evolution [26]–[28] and exhibits a general trend of increased complexity along the hierarchy of species [29], [30]. However, spermatogenesis process is sometimes similar between species with large evolutionary distance, such as cephalopods and rodents, and many developmental mechanisms involved in it seem to be conserved regardless of the taxonomic position [31]–[33]. In cephalopods spermiogenesis, it is common that perinuclear microtubule-based complexes analogous to mammalian manchette will also emerge as transitory structure and disappear from the cell after chromatin is completely condensed [34]–[39]. Researchers conclude that the progressive contraction of perinuclear microtubules and coordinated condensation of chromatin are two main determinants of successful nuclear morphogenesis in this taxon [3], [28], [40]–[43]. However, despite the attractive possibility that the molecular mechanism underlying sperm nuclear morphogenesis in cephalopods shares several crucial elements such as microtubules and motor proteins with that of rodents, whether and how motor protein like KIFC1 is associated with the cephalopod counterpart of manchette and participates in nuclear shaping as it does in rodents remains enigmatic. Elucidation of such functional mechanism in basal phyla would provide important clues concerning the evolution of sperm nuclear shaping machinery.

**Figure 1:**
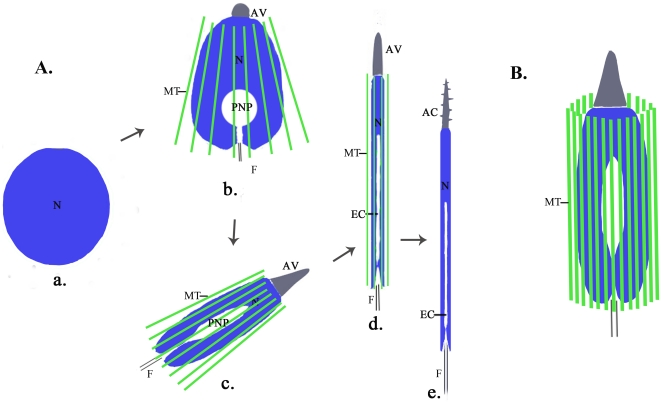
Diagrammatic representation of spermiogenesis in *O. tankahkeei* (diagram not to scale). (**A**) Morphological differentiation of the nucleus, acrosome, and flagellum is emphasized. In early spermatid (a), the nucleus is nearly round and other major organelles do not begin to differentiate; In intermediate spermatid (b), perinuclear manchette appears, the nucleus is slightly elongated and compacted and invaginated by a posterior nuclear pocket, a coalesced acrosomal vesicle is seen at the apical-most part, and the flagellar axoneme begins to assemble; In late spermatid (c), perinuclear manchette continues to closely wrap the nucleus, the nucleus is undergoing dramatic elongation and condensation and resembles a long spindle, the acrosomal vesicle grows in size and tapers forward, and the flagellum extends in length (not shown); The final spermatid (d) is almost identical to mature spermatozoon, perinuclear manchette begins to disassemble, the nucleus adopts a long cylindrical shape with endonuclear channel formed at its posterior end, the acrosomal vesicle becomes an extended cone-like structure and will undergo a final process of helicoidization, and the flagellum is very long (not shown); The mature spermatozoon (e) has a rod-like nucleus, a screw-shaped acrosome, and a slender flagellum. Perinuclear manchette completely disappears. N: nucleus; AV: acrosomal vesicle; AC: acrosome; F: flagellum; PNP: posterior nuclear pocket; EC: endonuclear channel; MT: manchette microtubule. (**B**) A perinuclear microtubules-based structure analogous to mammalian manchette is formed during sperm nuclear morphogenesis in *O. tankahkeei*. The microtubules are considered to run parallel to the long axis of the nucleus and wrap the nucleus. MT: manchette microtubule.

Spermatid differentiation in *Octopus tankahkeei* can be divided into several continuous stages based on the morphological features of major organelles (Figure 1A). During *O. tankahkeei* spermiogenesis, a manchette-analogous structure is also temporarily present in close proximity to the external nuclear membrane (Figure 1B) and has a role in the elongation and compaction of the developing nucleus [44]–[46]. The dramatic nature of the sperm nuclear shaping process in this organism enables it to be a good model system to investigate whether a cephalopod prototype of mammalian manchette-based and KIFC1-dependent sperm nuclear shaping machinery has evolved. We hypothesized that the *Octopus* version of rat KIFC1 will also participate in nuclear shaping process during *O. tankahkeei* spermiogenesis directly or indirectly. In this study, we detected the presence of a KIFC1-like protein from the testis of *O. tankahkeei* and provided novel observations on the dynamic localization of the protein and perinuclear microtubules in spermatogenic cells at different stages. Based on the results, we also sought to work out the functional models of the KIFC1-like protein with respect to the nuclear morphogenesis in *O. tankahkeei*.

## Materials and Methods

### Sampling

*O. tankahkeei* used in this study were purchased from local aquatic markets in Ningbo, China, from December, 2008 to November, 2009. In all, 30 male animals with various maturities were selected and maintained in sea water tanks with aeration. Following temporary maintenance, the animals were all anaesthetized on ice and dissected to collect the testes and other tissues. The obtained tissues were then subjected to corresponding treatments in different experiments.

### Antibodies

Details of antibody preparation were provided by Yang and Sperry (2003). Briefly, the KIFC1 antiserum was raised in rabbits by the Antibody Research Center, Shanghai Immune Biotech Co., Ltd (Shanghai, China) against a unique peptide (QGKAASGASGRAAAIA) of KIFC1 highly conserved among its homologues and producing negligible homology with other recorded sequences in the GeneBank database. Then, the polyclonal antibody capable of recognizing KIFC1 and KIFC1-like proteins was affinity purified with the Sulfolink Kit (Pierce, Rockford, IL, USA) according to the manufacture's instruction. Other antibodies used in this study were purchased from commercial suppliers and detailed in respective sections.

### Western Blot

We extracted total protein from the testis, liver and muscle of sexually mature *O. tankahkeei*, respectively, in light of the protocol described by Yang and Sperry (2003). In brief, fresh tissues were homogenized in buffer containing protease inhibitors and centrifuged at 14000 rpm at 4°C for one hour. Then the high-speed supernatant (HSS) fraction was obtained. After determination of protein concentration in the HSS, samples with equivalent total protein were separated by polyacrylamide gel electrophoresis in 10% acrylamide gels and electrophoretically transferred to polyvinjylidene difluoride (PVDF) membranes (Bio-Rad). Proteins were incubated on the membrane with KIFC1 polyclonal antibody. The blots were detected with horseradish peroxidase-conjugated donkey secondary antibody diluted 1∶20000 in TBST (100 mM TRIS pH 7.5, 150 mM NaCl, 0.1% Tween20) and developed with enhanced chemiluminescent reagents (Amersham Pharmacia Biotech).

### Immunofluorescence (IF)

Testes were dissected from animals with various maturities and immersion fixed overnight in 4% paraformaldehyde- phosphate buffered saline (PBS) (pH 7.4) at 4°C. Then they were placed in 0.5 M sucrose-PBS (pH 7.4) at 4°C until they sunk, followed by embedding in OCT compound at −20°C. The tissues were cut into 7-µm frozen sections and transferred to slides. After drying at 37°C, the sections were treated with 0.2% Triton X-100 in PBS (pH 7.4) for 15 min at room temperature. The tissue was then blocked in 6% bovine serum albumin (BSA) in TBST (20 mM Tris, pH 7.5, 154 mM NaCl, 2 mM EGTA, 2 mM MgCl_2_, 0.1% Triton X-100) for 1 hour at room temperature. Testis section was first incubated with KIFC1 antibody and then with Texas Red-conjugated goat anti-rabbit IgG (1∶200 dilution; Jackson ImmunoResearch Laboratories, West Grove, PA, USA) and FITC-conjugated anti-α tubulin (1∶150 dilution; Sigma, St. Louis, MO, USA) for 1 hour. After 3 times' rinse in TBST, the sections were stained with DAPI and mounted. The location of respective substance in the cells was observed under a Zeiss laser scanning confocal microscope (LSM 510; Carl Zeiss, Thornwood, N.Y., USA) fit with appropriate filters and images were captured with an internal camera. Omissions of primary or secondary antibody in parallel experiments were set as negative controls (data not shown).

### Immunogold Electron Microscopy (IEM)

IEM was carried out according to the protocol detailed by Yang and Sperry (2003) with some modification. Testes were obtained from the animals and fixed in 4% paraformaldehyde and 0.1% gluteraldehyde in PBS (pH 7.4). After dehydration by ethanol series and pre-embedding in LR White (Ted Pella, Inc., Redding, Calif., USA), the tissues were transitioned to gelatin capsules containing fresh embedding media which was then polymerized at 50°C for 24 hours. Samples were then sectioned at 130-nm on a Reichert ultramicrotome (Labequip, Markham, Ontario, Canada). The sections were blocked in 5% egg albumin for 2 h before incubation with KIFC1 polyclonal antibody (diluted 1∶50 in 0.04 M PBS, pH 7.4, 0.5% BSA) for 1 h at room temperature and then with goat anti-rabbit IgG conjugated to 25 nm colloidal gold particle (Amersham Pharmacia Biotech, Piscataway, N.J., USA) (diluted 1∶20 in 0.04 M PBS, pH 8.2, 0.5% fish gelatin) for 1 h at room temperature. Following appropriate wash and rinse, the sections were air dried and counterstained with 7.5% uranyl acetate for 40 min and lead citrate for 2 min. Finally, the stained sections were observed and photographed under a transmition electron microscope. Omissions of primary or secondary antibody in parallel experiments were set as negative controls (data not shown).

## Results

### KIFC1-like protein is present in various tissues of *O. tankahkeei*

**Figure 2:**
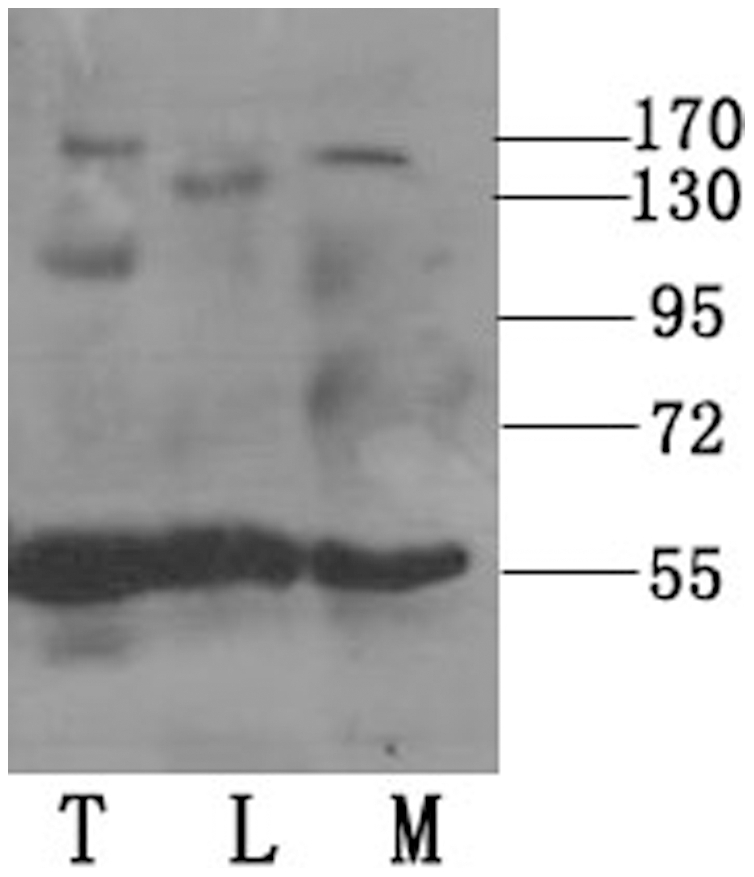
Detection of the KIFC1-like protein by Western Blot. The KIFC1-like protein was present in the testis (T), muscle (M) and liver (L) of *O. tankahkeei*. The protein migrated to a position on the membrane corresponding to a molecular weight of slightly above 55 kDa. No obvious difference of protein expression level was discerned from the result. Molecular weight marker (kDa) was labeled on the right.

The motor protein KIFC1 was initially identified in mouse embryonic brain. Thereafter its presence in other tissues including liver, ovary, lung and spleen was also reported [21]. Further molecular research showed that two similar molecular motors KIFC1 and KIFC5 were associated with multiple microtubule complexes in male germ cells, including the meiotic spindle, the manchette, and the flagellum [47], with important roles. In this experiment, an unidentified protein from the testis, liver and muscle of mature *O. tankahkeei* was detected in Western blot with an antibody raised against a unique antigenic peptide of KIFC1. The putative KIFC1-like protein migrated to a position on the membrane corresponding to a molecular weight of 55–60 kDa (Figure 2). Equivalent quantity of protein from each tissue was loaded in the experiment, and no obvious difference of protein expression level among the tissues was discerned from the result. The presence of this motor protein in these tissues is consistent with the tissue distribution pattern of KIFC1 in mouse [47] but not with that in the Chinese mitten crab [48].

### KIFC1-like protein is not expressed at early stages of spermatogenesis

**Figure 3:**
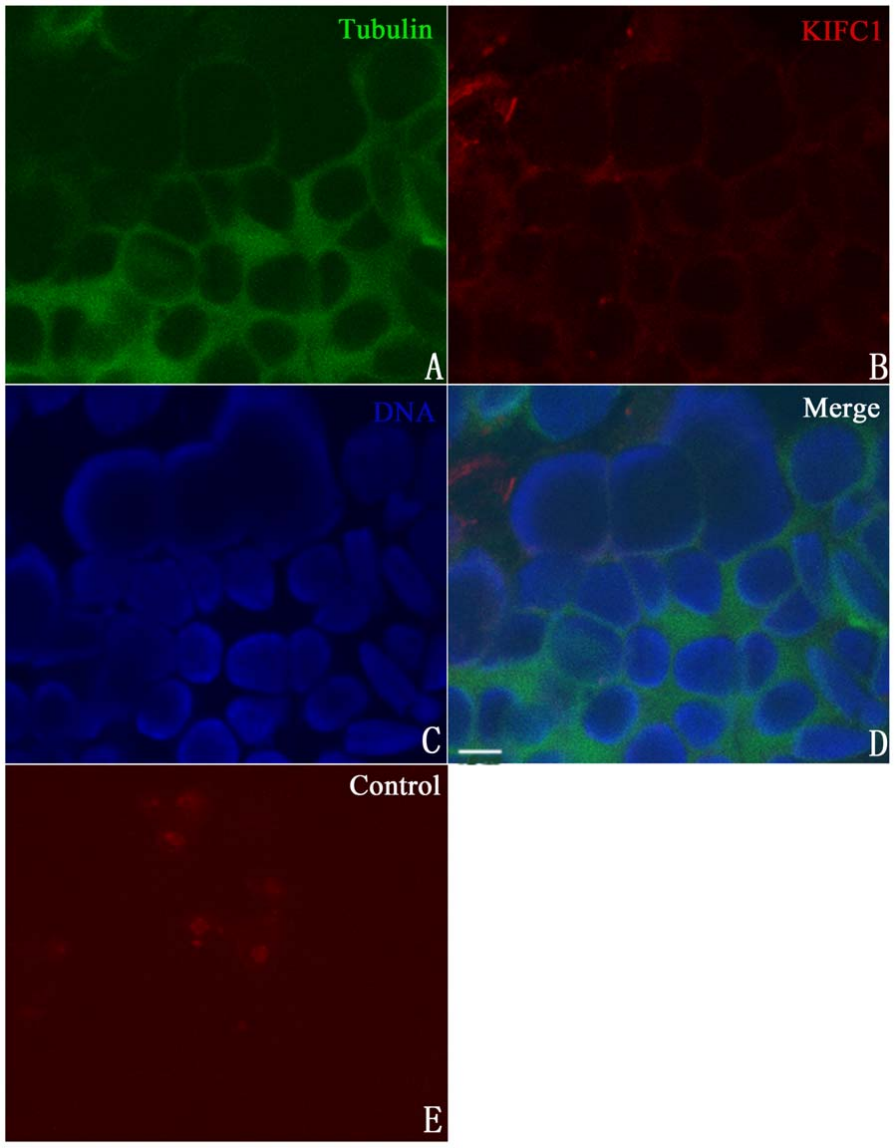
Absence of the KIFC1-like protein at early stages of spermatogenesis in *O. tankahkeei*. The protein was immuno-detected with KIFC1 antibody and Texas Red conjugated secondary antibody. Tubulin was localized using FITC-conjugated anti-tubulin antibody. DAPI was used to stain DNA. (**A**) Microtubules (green signal) were randomly scattered in the cytoplasm. (**B**) The KIFC1-like protein (red signal) was not expressed at the moment. (**C**) Nuclei staining (blue signal) of spermatogonia or spermatocytes. (**D**) A merge of the above three pictures. (**E**) Omission of antibody to KIFC1 was set as a negative control. Scale bar equals 5 µm.

To determine the dynamic distribution of the KIFC1-like protein during spermatogenesis, we analyzed frozen sections of testis by Immunofluorescence and Immunogold Electron Microscopy. The early stages of spermatogenesis refer to the period in which no significant morphological change occurs to the cell. Spermatogenic cells at the early stages include spermatogonia and spermatocytes. At the onset of spermatogenesis, the shape of the cell is nearly round with many mitochondria in the cytoplasm near the Golgi apparatus, and chromatin will gradually aggregate into clusters in the nucleus [45]. Based on previous morphological investigation and the size of cell, we were able to recognize the expression and distribution of the KIFC1-like protein in spermatogonia or spermatocytes (Figure 3B). Microtubule was randomly scattered in the cytoplasm (Figure 3A), and expression of the KIFC1-like protein was hardly detectable at this moment (Figure 3B). This phenomenon was in good agreement with the situation in rat where KIFC1 did not appear at the beginning of spermatogenesis [43].

### KIFC1-like protein initiates its expression in early spermatid

**Figure 4:**
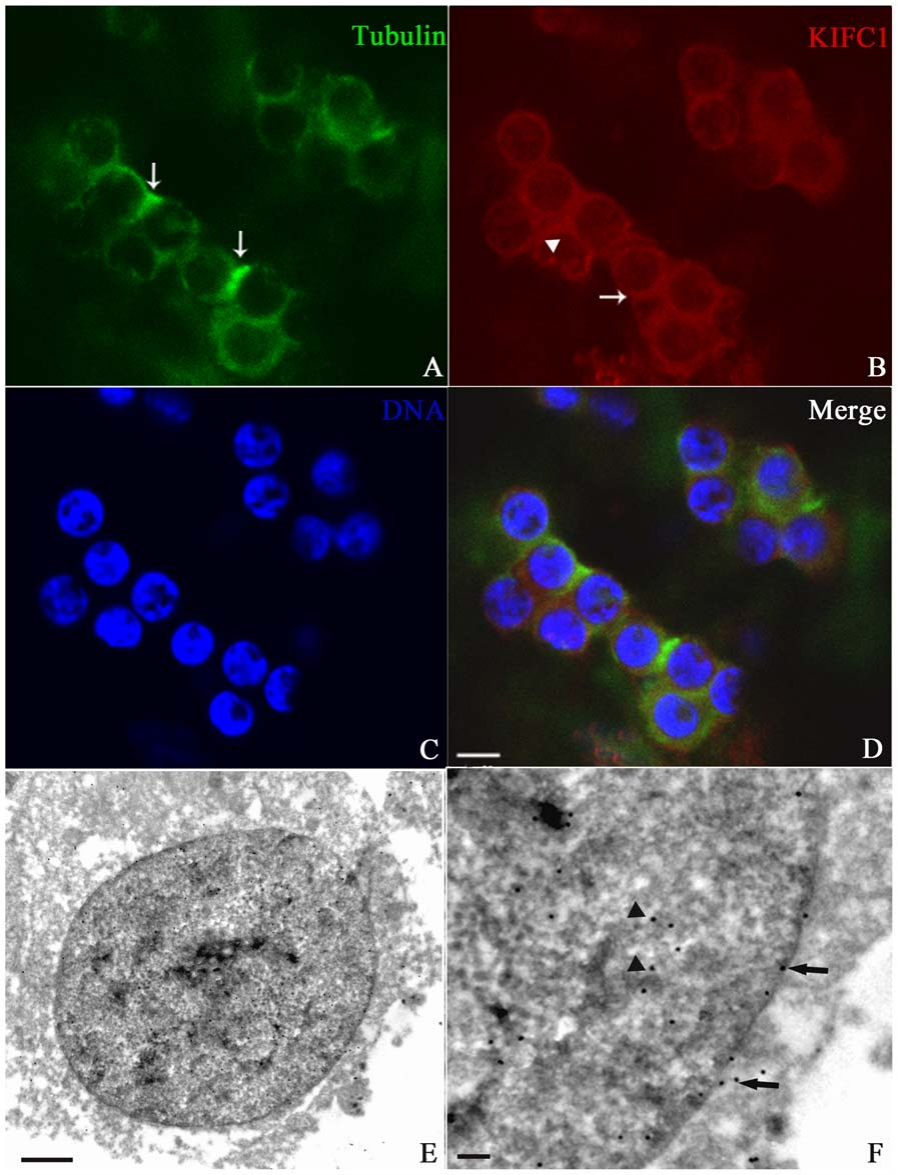
Distribution of the KIFC1-like protein and microtubules in early spermatid in *O. tankahkeei*. The KIFC1-like protein and microtubules are visualized by IF and IEM. (**A**) Microtubules seemed preferentially arranged near the basal zone of the spermatid head (arrow) where the manchette-like structure began to assemble. The green signal displayed varying intensity at different places and was not continuous around the nucleus. (**B**) Elevated expression of the KIFC1-like protein was initiated. The signal was apparent in vicinity to the nuclear envelope (arrow) and also present in the nucleus with lower intensity (arrowhead). (**C**) Nuclei staining of early spermatid. (**D**) A merge of the above three pictures. (**E**, **F**) The gold particles were located near the nuclear membranes (arrows) and in the nucleus (arrowheads). F is a higher magnification of an area in E. Scale bar is 5 µm in (A–D), 1 µm in (E) and 0.25µm in (F).

The early spermatid is similar to the secondary spermatocyte in size and shape, but its chromatin becomes more evenly distributed within the nucleus (Figure 4C). Electron Microscopy results showed the chromatin was transformed into homogeneously packaged granules with some large electrondense aggregates (Figure 4E). The profile of chromatin condensation at this stage is very similar to that in other cephalopod species. At this stage, microtubules seemed preferentially arranged in a confined region of spermatid (Figure 4A, arrows), which was in agreement with a report on another cephalopod *S. officinalis* proposing that perinuclear microtubules originated from the basal zone of spermatid head and grew toward the acrosome [5]. The green signal displayed varying intensity at different places and was not continuous around the nucleus, suggesting the manchette-like perinuclear structure was not yet formed (Figure 4A). Meanwhile, elevated expression of the KIFC1-like protein was already initiated. The signal was apparent in the cytoplasm (Figure 4B, arrow) and in vicinity to the nuclear envelope (Figure 4F, arrows). It was also present in the nucleus with lower intensity (Figure 4B, F, arrowheads), indicating some functions of the protein in the nucleus. In a previous study, the molecular motor KIFC1 was mainly located near the Golgi apparatus in early spermatids and enriched in the nucleus of round spermatids of rat [24]. This distribution pattern at the beginning of spermiogenesis was not observed in *O. tankahkeei*.

### KIFC1-like protein associates with perinuclear microtubules and nuclear envelope in spermatids undergoing dramatic elongation and condensation

**Figure 5:**
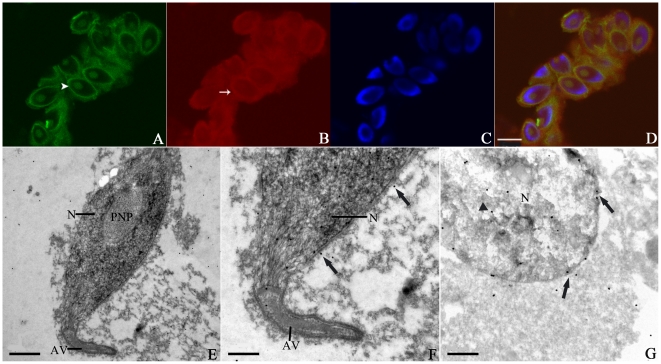
Localization of the KIFC1-like protein and microtubules in intermediate spermatid in *O. tankahkeei*. The distribution of KIFC1-like protein and microtubules are observed by IF and IEM. (**A**) Microtubules were tightly attached to the nuclear periphery (arrowhead). (**B**) The KIFC1-like protein was abundantly expressed and mainly located around and in the nucleus (arrow). (**C**) Nuclei staining of intermediate spermatid. (**D**) A merge of the above three pictures. (**E**, **F**) Longitudinal sections of the spermatid. Some of the gold particles were located in proximity to the nuclear perimeter (arrows) corresponding to the growing manchette-like structure. F is a higher magnification of an area in E. (**G**) Transverse section of the spermatid. The gold particles were clearly seen near the nuclear envelope (arrows) as well as in the nucleus (arrowhead). Scale bar is 5 µm in (A–D), 1 µm in (E) and 0.5 µm in (F and G). AV: acrosomal vesicle. N: nucleus. PNP: posterior nuclear pocket.

**Figure 6:**
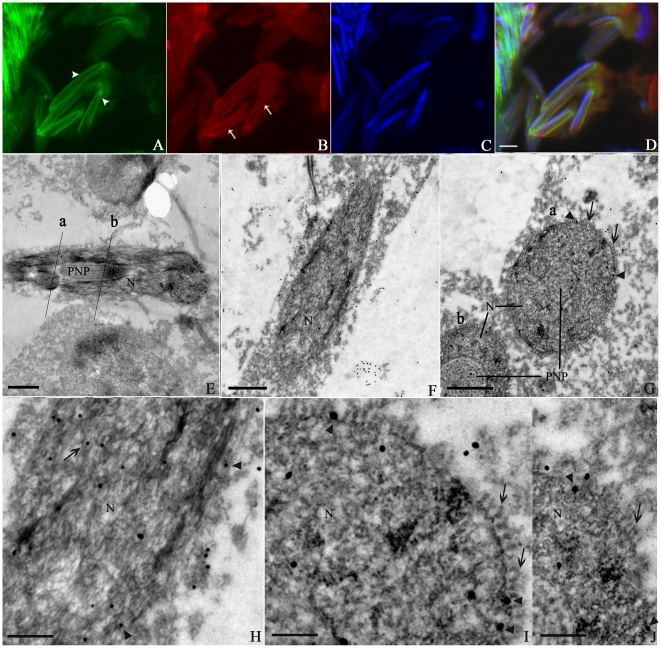
Localization of the KIFC1-like protein and microtubules in late spermatid in *O. tankahkeei*. The IF and IEM methods were hired to detect the expression and localization of KIFC1-like protein and microtubules at the late spermatid stage. (**A**) The manchette-like structure was seen encircling the nucleus (arrowheads). (**B**) The KIFC1-like protein was still mainly located around and in the nucleus, but the expression level was relatively decreased as the signal intensity declined (arrows). (**C**) Nuclei staining of late spermatid. (**D**) A merge of the above three pictures. (**E**, **F**, **H**) Longitudinal sections of a segment of the spermatid. The gold particles were located in proximity to the nuclear periphery (arrowheads) as well as in the nucleus (arrow). Cross sections at two positions labeled as “a” and “b” were presented in G. H is a higher magnification of an area in F. (**G**, **I**, **J**) Transverse sections of the spermatid. Some gold particles (arrowheads) were distributed between the nuclear envelope and the manchette-like perinuclear structure (arrows) which exhibited a transverse profile of a circle of holes closely attached to the nuclear periphery. I and J are higher magnifications of an area of “a” and “b” labled in G, respectively. Scale bar is 5 µm in (A–D), 1 µm in (E-G), 0.5 µm in (H) and 0.25 µm in (I and J). N: nucleus. PNP: posterior nuclear pocket.

The spermatids will undergo a stage of dramatic elongation and compression before acquisition of a long cylindrical shape. Intermediate spermatid and late spermatid belong to this stage. During this stage, the nucleus of spermatid begins to elongate and compress and eventually resembles a long spindle, with the narrow end adjacent to the emerging acrosome and the opposite end close to the centriole [45]. The endonuclear pocket at the caudal end can be seen in the nucleus (Figure 5C). At the apical part of spermatid, the acrosome continues to differentiate (Figure 5E, F). Immunofluorescence and Immunomicroscopy results clearly demonstrated that microtubules were tightly attached to the nuclear periphery throughout this period (Figure 5A; 6A, G, I), consistent with previous reports claiming it was during this stage that perinuclear microtubules will gradually form a transient structure analogous to mammalian manchette [45]. However, unlike the manchette that anchors its constituting microtubules through the nuclear ring and encompasses only the caudal half of nucleus, perinuclear microtubules in this species seem to assemble without presence of a nuclear ring and cover the entire surface of the nucleus (Figure 5A; 6A). Meanwhile, the KIFC1-like protein was abundantly expressed and mainly located around the nuclear envelope and in the nucleus (Figure 5B, G; 6B, G, H). Colocalization of the KIFC1-like protein with the assumed manchette-like microtubule structure in close proximity to the nuclear boundary was readily observed (Figure 5D, F; 6D, I, J), demonstrating association of the KIFC1-like protein with perinuclear microtubules and nuclear envelope and supporting the speculation that the motor protein is engaged in bridging perinuclear microtubules to the nucleus.

### KIFC1-like protein is enriched at one end of final spermatid

**Figure 7:**
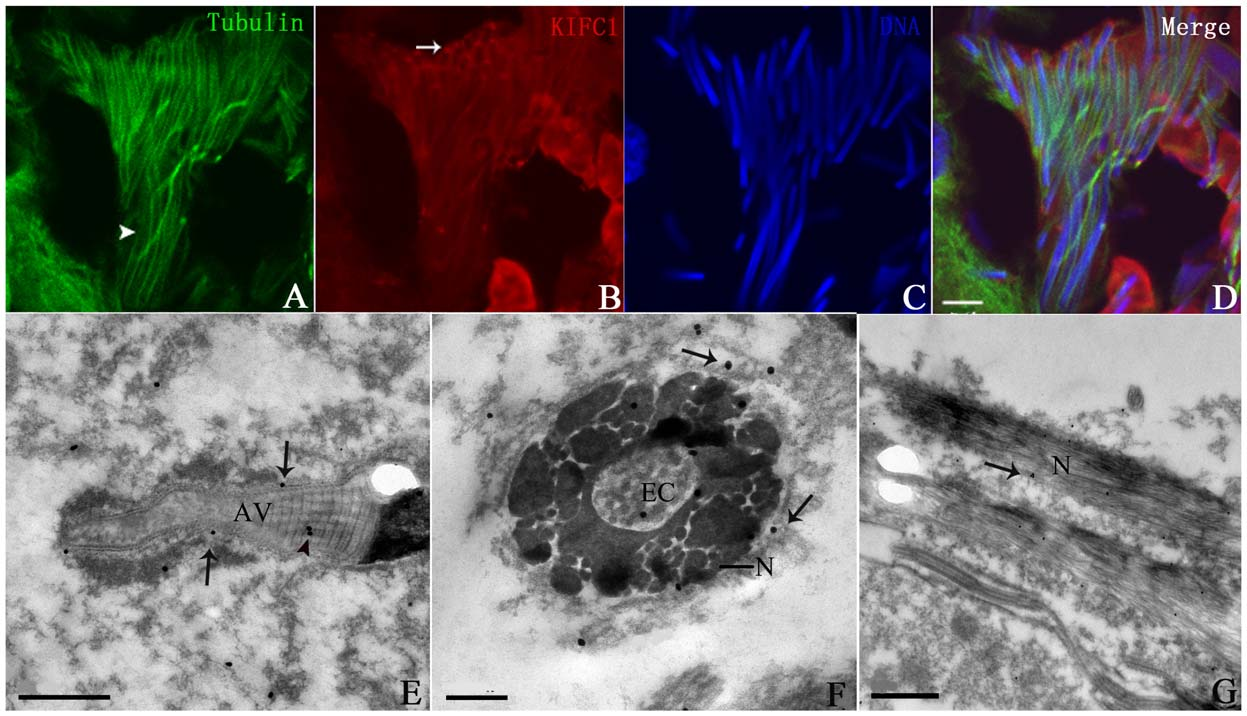
Localization of the KIFC1-like protein and microtubules in final spermatid in *O. tankahkeei*. The location of KIFC1-like protein and microtubules were checked by IF and IEM. (**A**) Microtubules were still visible in the cell but the manchette-like perinuclear structure was not so rigid around the nucleus (arrowhead). (**B**) Signal intensity of the KIFC1-like protein greatly decreased, indicating its expression level significantly dropped. The signal around and in the nucleus was not as apparent as seen in previous stages. Most of the protein would concentrate like a dot at one end (arrow) of the spermatid probably corresponding to the caudal region where residual cytoplasm was disposed. (**C**) Nuclei staining of final spermatid. (**D**) A merge of the above three pictures. (**E**) Longitudinal section of the maturing acrosome. The gold particles were present in the inner striated structure (arrowheads) and near the helical surface (arrows). (**F**) Transverse section of the spermatid. The manchette-like perinuclear microtubules were absent. The gold particles were seen near the nuclear envelope (arrows) and in the nucleus (arrowhead) with low density. (**G**) Longitudinal section of the spermatid. The gold particles were sparse in the cell, implying significant decline of protein expression. Scale bar is 5 µm in (A–D), 1 µm in (E and G), and 0.25 µm in (F). AV: acrosomal vesicle. N: nucleus. EC: endonuclear channel.

In final spermatid, major organelles are going through the last period of development leading to maturation. The acrosome is completing the final process of helicoidization, the nucleus has adopted a long cylindrical shape almost identical to mature sperm, and the perinuclear microtubules will gradually disassemble until disappearing in mature sperm. Residual cytoplasm is translocated to the caudal end of spermatid to be eliminated. According to IF and IEM micrographs, microtubules were still visible in the cell but the manchette-like perinuclear structure was not so rigid around the nucleus (Figure 7A, arrowhead). It was also noticed that signal intensity of the KIFC1-like protein greatly decreased (Figure 7B, F, G), indicating its expression level relatively declined in final spermatid. The signal around and in the nucleus was just detectable and not as apparent as seen in previous stages. On the other hand, it seemed that most of the protein signal would concentrate like a dot at one end of the spermatid (Figure 7B, arrow). Judging from the relative signal intensity in the anterior region (Figure 7E), this end probably corresponded to the posterior end where residual cytoplasm was disposed, which unveiled the motor's directed locomotion from the head to the caudal part of spermatid.

## Discussion

Spermatogenesis is one of the most fundamental developmental programs related to animal sexual reproduction [33]. Many cellular transformations take place during this process. Among them is morphogenesis of the nucleus, or nuclear shaping. The nucleus of mature spermatozoon varies dramatically among different taxonomic groups, so does the way the final morphology of nucleus is achieved [28]. Therefore, comparison of nuclear morphology and the molecular mechanism for nuclear shaping could be a potential indicator of sperm evolution.

The morphological differentiation of spermatid in cephalopods is in many aspects similar to that in higher organisms such as mammals and birds despite the evolutionary distance between them [11], [43], [49]. The mature spermatozoa of these groups of animals possess three major functional organelles, namely an anterior acrosome, a condensed and elongated nucleus, and a slender motile flagellum. In mammals, it was proposed that among other cellular factors involved in nuclear shaping, cytoskeleton network and associated motor proteins played pivotal roles in this process [50]–[56]. Particularly, a microtubule-based perinuclear structure transiently present during spermiogenesis, or the manchette, and a C-terminal kinesin motor KIFC1 were believed to play essential roles in the successful elongation and compaction of the nucleus [7], [19], [25], [57], [58]. The manchette allegedly serves as a platform for placement of regulatory proteins at appropriate times and sites to control key events in spermiogenesis [7], [15], [59]. Irregularities of manchette microtubules in spermatids and spermatozoa induced by chemical treatment or specific mutation of β-tubulin gene will result in nuclear abnormalities [57], [60]. The manchette-binding molecular motor KIFC1 would interact with a nucleoporin-containing complex on the nuclear membrane and contribute to nuclear shaping [25]. KIFC1 was also implicated in vesicle transport from Golgi apparatus to growing acrosome. By contrast, spermatogenesis in cephalopods as a whole has only been investigated from the perspective of ultrastructural observation [3], [35], [61] and the molecular mechanism governing the intriguing cytological changes such as nuclear shaping is barely explored.

Spermatogenesis in *O. tankahkeei* is a good model system to examine whether the role of KIFC1 and microtubules in nuclear shaping is evolutionarily conserved. In our experiment, the antibody specific to a unique and conserved peptide of KIFC1 required for its intracellular targeting was able to detect a protein in Western Blot, suggesting the existence of a KIFC1-like protein in *O. tankahkeei*. The protein was present not only in the testis but also in other tissues, indicating the potential non-reproductive functions of this motor in somatic cells of this organism. This finding echoes existing literature documenting that coordinated activity of KIFC1 and KIF5B is essential for motility and fission of early endocytic vesicles in mouse liver [62].

IF and IEM analysis disclosed the dynamic localization of the KIFC1-like protein and microtubules along the progression of spermatogenesis. At the beginning, the protein was not apparently expressed, and microtubule was randomly distributed in the cytoplasm. When it comes to the early spermatid, the protein began to be expressed and partly colocalize with cytoplasmic microtubules. Similarly, in intermediate and late spermatids where pronounced nuclear elongation and condensation took place and a microtubules complex was present in vicinity to the nucleus, expression of the protein was maintained at a high level and most protein signal was detected around the nucleus and overlapped with perinuclear microtubules. Furthermore, the protein appeared to accumulate at caudal end of final spermatid, suggesting its directed movement from the head to the caudal region. These data collectively provided evidence for our hypothesis regarding association of the KIFC1-like motor with the cephalopod counterpart of mammalian manchette and their involvement in nuclear shaping.

Formation of perinuclear microtubules complex is very common in cephalopod spermatogenesis, and a number of reports have proposed that this complex is engaged in some morphogenesis events [3], [28], [34], [63]. For example, in *Sepia officinalis*, it is observed that perinuclear microtubules begin to assemble in early spermatid and will then interact with condensing chromatin through establishing contact with the external nuclear membrane [28]. Sperm nuclear morphogenesis in *S. officinalis* is considered to be dictated by the interaction between perinuclear microtubules and condensing chromatin. This type of interaction is confirmed in *Octopus* and *Eledone* as well [3], [63], [64]. Nonetheless, an important question remains to be addressed: How are perinuclear microtubules linked to nuclear membranes and further to components in the nucleus such as chromatin? In other words, if perinuclear microtubules could interact with chromatin through nuclear membranes, there should be a bridge between them. Here, during *O. tankahkeei* spermiogenesis, the KIFC1-like motor protein was distributed largely around the nucleus and partly colocalized with the perinuclear manchette-like structure when the nucleus was longitudinally elongated and laterally compressed, enhancing the possibility that the KIFC1-like protein is able to link perinuclear microtubules to the nucleus and participate in nuclear morphogenesis.

When we ponder over how the KIFC1-like protein and perinuclear microtubules are able to assist in transforming nuclear shape, an essential structure inside the nucleus, the nuclear lamina, has to be taken into account. The nuclear lamina is a layer of fibrillar network composed of lamins and lamin-associated proteins near the inner nuclear membrane [65], [66]. It is involved in a lot of nuclear activities including chromatin organization, nuclear migration, and cell differentiation [65], [67]. Genetic studies on some model organisms have demonstrated that the nuclear lamins are responsible for maintaining nuclear shape [68]–[71]. In addition, relationship of the nuclear lamina with spermiogenic chromatin and nuclear morphogenesis has also been reported in an octopod *Octopus vulgaris* [42]. Therefore, chances are that any significant change of nuclear shape is correlated with active reorganization of the nuclear lamina.

**Figure 8:**
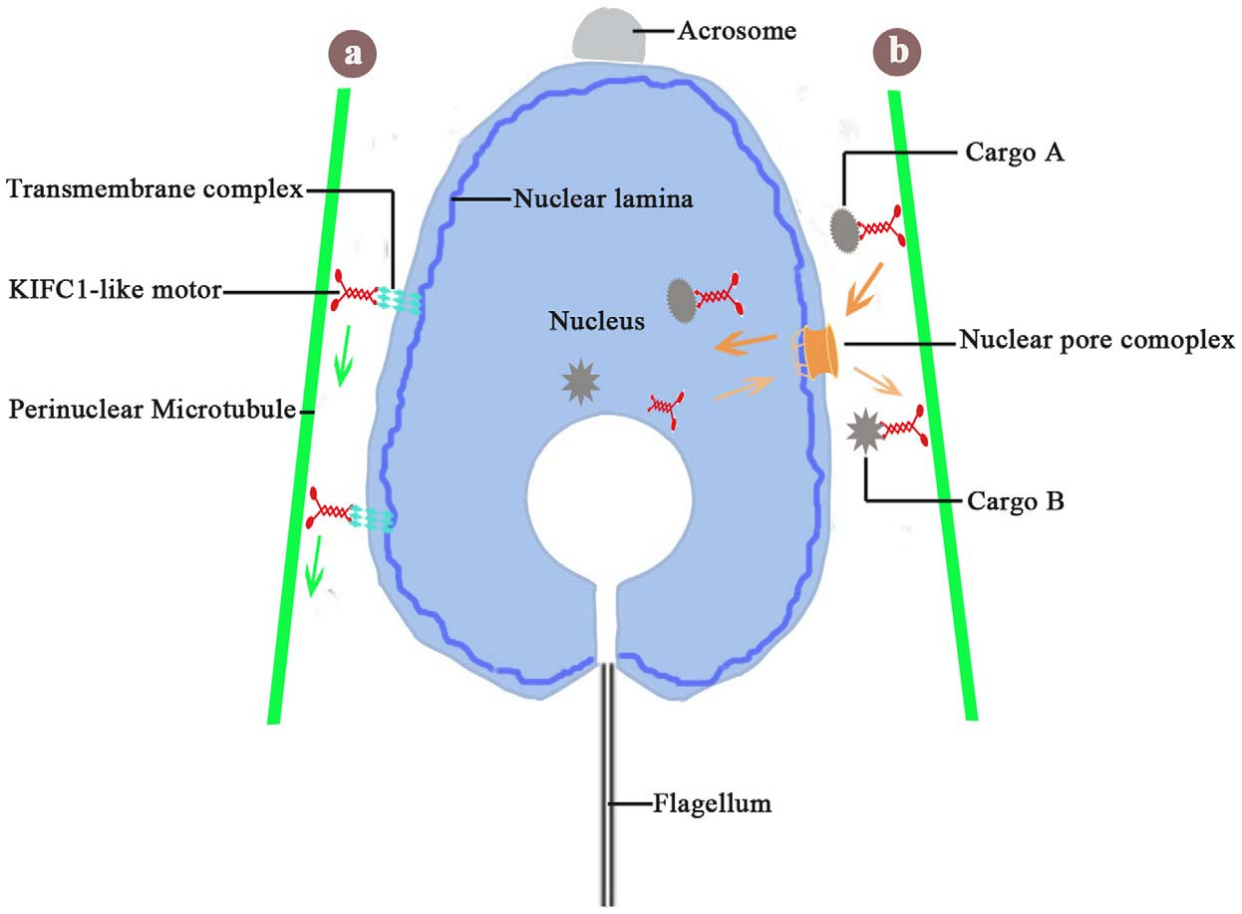
Two possible functional models of the KIFC1-like protein in sperm nuclear morphogenesis in *O. tankahkeei*. (**a**) The manchette-like perinuclear microtubules are connected to the nuclear lamina inside the nucleus through association of the KIFC1-like protein with some multi-molecular complex (transmembrane complex) scanning the nuclear membranes and reaching the nuclear lamina. The KIFC1-like protein moving towards the caudal end of spermatid will transmit the motor-generated force to the nucleus and facilitate mechanical rearrangement of the nuclear lamina and other endonuclear components and eventually elongation and condensation of the nucleus. (**b**) The KIFC1-like protein is able to carry some structural and functional elements in vicinity to the nuclear perimeter and shuttle across the nuclear pore complex to enter or exit the nucleus via association with the nucleocytoplasmic trafficking machinery. As a result, it will assist in the import (cargo A) and export (cargo B) of a specific group of cargoes probably involved in the regulation of nuclear activities such as chromatin condensation.

A possible functional model of the KIFC1-like protein is that it will interact with some multi-molecular complex scanning the nuclear membranes and stretching to the nuclear lamina inside the nucleus (Figure 8a). According to this model, the KIFC1-like protein binds to perinuclear microtubules via its motor domain at the C-terminal and attaches to the trans-membrane molecular complex by its cargo domain at the N-terminal, while the nucleoplasmic face of the trans-membrane complex is associated with the nuclear lamina. In this way, the manchette-like perinuclear structure is connected to the nuclear lamina, thus enabling the motility of perinuclear microtubules and motor proteins to influence the activity of the nuclear lamina. The endonuclear chromatin is continuously condensed and packaged during nuclear morphogenesis, making the nucleus not so rigid and somewhat flexible. That means the KIFC1-like protein moving towards the caudal end of spermatid will transmit the motor-generated force to the nucleus and, instead of bringing about translocation in position, facilitate mechanical rearrangement of the nuclear lamina and other endonuclear components and eventually elongation and condensation of the nucleus.

Precedents for transmembrane complex linking cytoskeleton to endonuclear structures have made the proposed model quite promising. In several cell types, a protein complex composed of two subunits, a SUN-domain-containing protein and a KASH-domain-containing protein, is discovered spanning the double membranes of the nuclear envelope [72], [73]. The cytoplasmic end of the SUN-KASH complex is associated with cytoskeleton network directly or via molecular motors such as dynein and kinesin, while the nucleoplasmic end of it interacts with the nuclear lamina [73]. Therefore, the SUN-KASH complex serves as a bridge between cytoskeleton and nucleo-skeleton and plays critical roles in numerous cellular processes such as nuclear migration and cytokinesis [72], [74]. We are not sure whether a SUN-KASH complex is present in spermatic cells of octopods. However, it is still conceivable that a similar strategy for nucleo-cytoskeletal connection may have evolved in this lineage, supporting the potential existence of the multi-molecular transmembrane complex in model a. This model would also provide a reasonable answer for the aforementioned question regarding how interaction between perinuclear microtubules and endonuclear chromatin is established in cephalopods, yet we need more in-depth experiments to challenge and testify this answer.

Another possible functional model of the KIFC1-like protein in nuclear shaping is based on its constant presence in the nucleus during this process and previous studies in rat claiming that importin β, one crucial component of the nucleocytoplasmic transport machinery, was co-immunoprecipitated with KIFC1, confirming the interaction between importin β and KIFC1 and the potential involvement of KIFC1 in this pathway [25]. Nuclear morphogenesis is a dynamic process requiring successive and timely transition of structural and functional elements both outside and within the nucleus [7], [75], [76]. In this model (Figure 8b), the KIFC1-like protein in *O. tankahkeei* is able to carry some structural and functional elements (cargo A and cargo B) in vicinity to the nuclear periphery and shuttle across the nuclear pore complex to enter or exit the nucleus via association with the nucleocytoplasmic transport machinery. As a result, it also performs some functions independent on microtubules and will assist in the import and export of a specific group of cargoes probably involved in the regulation of nuclear activities such as chromatin condensation. Supporting this idea, the KIFC1-like protein was found within the nucleus and on the nuclear membranes even before the manthette-like structure took shape in early spermatid, and it was also constantly present in the nucleus during nuclear elongation and condensation. These phenomena are consistent with a presumed nucleocytoplasmic-trafficking-related role of the KIFC1-like protein in model b.

It is no doubt that more concrete evidences are needed to disprove and modify the two proposed models before any conclusion could be made on the precise functional mechanism of the KIFC1-like protein. However, the presence of a KIFC1-like protein and perinuclear microtubules along with their association during nuclear morphogenesis could be interpreted as molecular and cellular traces of an ancient nuclear shaping machinery, and the current study strongly suggests that an ancestral prototype of mobilization of manchette and the kinesin motor KIFC1 in sperm nuclear morphogenesis has already evolved in cephalopods, or at least in octopods. This further highlights the possibility that a few core components of sperm nuclear shaping machinery remain conserved across large evolutionary distances.
